# A State Board of Health for Texas

**Published:** 1896-06

**Authors:** 


### EDITORIAL DEPARTMENT.

F. E. DANIEL, M. D., Editor.
S. E. HUDSON, M. D., Managing Editor.
A. J. SMITH. M. D., Galveston, Associate Editor.

EDITORIAL STAFF:

PROF. J. E. THOMPSON, M. D., Texas Medical College, Galveston; Surgery.
PROF. WM. KEILLER, M. D., Texas Medical College, Galveston; Obstetrics and Gynecology.
PROF. DAVID CERNA, M. D., Texas Medical College, Galveston; Therapeutics.
PROF. A. J. SMITH, M. D., Texas Medical College, Galveston; Medicine
DR. R. H. L. BIBB, City of Mexico; Foreign Correspondent.

Official organ of the West Texas Medical Association, the Houston District Medical Association, the Austin District Medical Society, the Galveston County Medical Society, and several others.

### A STATE BOARD OF HEALTH FOR TEXAS.

Lest the JOURNAL should be thought opposed to a State Board of Health because of its pronounced opposition to the wild vagary proposed by Dr. McLaughlin—to "memorialize the Legislature to transfer the expense of State quarantine to the general government," we take occasion to point to its record, dating back as far as 1882, when we first began to urge upon the Legislature the advisability of creating a State Board of Health. The JOURNAL has advocated first, last and all the time, building upon the present health system, believing it would be more expedient to have other duties added to those of State Health Officer, and giving him the necessary assistance, than to undertake the organization of a board *de novo;* and in 1885 the writer prepared a bill to this end, and it was presented, but failed to pass. This bill had the approval of the State Health Officer and of the Legislative Committee of the State Medical Association. It provided for a chemist and a Secretary, the duties of the latter to be—in addition to the clerical duties of the office, the compilation, preservation and publication of vital statistics; the State Health Officer to be ex-officio President of the Board.

We believe now, as we did then, that legislation in this direction will be easier to obtain than an act which will wipe out existing methods and create a board in *toto.* The Legislature has shown itself reluctant to meddle at all with the subject, being apparently satisfied with the results. They have observed the remarkable exemption of the State from infectious diseases, and are disposed to let well enough alone. It is well enough, so far as it goes, but it does not go far enough. The profession appreciate this, note the lack of other features, but so far, have failed, somehow, to impress upon the Legislature that a bacteriologist, a chemist and a Secretary are a necessity. When this can be done I apprehend there will be little difficulty in getting the required legislation to complete the system as above outlined.

In our last we said, "A State Board of Health is a popular idea, but we are pursuaded that the profession do not understand the situation." What we referred to was the failure in years past to secure the passage of any act by the Legislature that conflicts with the existing system. We thought, and we think now, that there is little encouragement to repeat a request so often refused; and we believe our only chances of success lie in the direction above indicated. Dr. McLaughlin's recent pronunciamento—in which he argues that a bob-tailed board of health of several members—i. e.—a board minus quarantine—would be less expensive to the State than the present system, has perhaps confused the subject in the minds of his readers, and especially with those who have been reading his articles in the *Texas Sanitarian;* for, no longer ago than February, 1895, he was advocating precisely what we here advocate—building on the one man system—the only difference between us then being—that the editor is for retaining the chief feature, quarantine, and he for getting rid of it; now he is for a board of several members. In the February, 1895, number, *Texas Sanitarian* (page 166) Dr. McLaughlin said: "We believe, and in this belief we differ from many others, that a 'one man power' representing the power of the State in matters of public health is better, and will do better service for the State than will a board with several members." * * * The doctor has apparently abandoned his platform of February, 1895. We stand squarely upon it, as we did in 1885, and have ever done,—differing with Dr. McLaughlin only on the one point mentioned; and we insist that if Texas is ever to have a State Board of Health, the above plan will not only be best, and will be more economical, and we are more likely to get it. * * * Enlarge the duties of the State Health Officer and give him the necessary officers and assistants—a bacteriologist, a chemist and a Secretary, and define the duties of each, and Texas will then have a board of which any State may be proud.

The reduction in the operating expenses of quarantine that has taken place under the present administration would be fully equal to the additions contemplated. Under former administrations the annual appropriation was $45,000. Under existing laws it is $33,000, and all of that amount was not expended last year. The saving thus effected would be more than sufficient to pay for the added functions without the pretext made by Dr. McLaughlin. The population of the State is rapidly increasing, and it is to be expected that the State expenses will increase *pari pasu* in all departments. We repeat, there is no necessity for the step proposed by Dr. McLaughlin.

